# Safety and efficacy of ultrasound guided corticosteroid injections into temporomandibular joints in children with active juvenile idiopathic arthritis

**DOI:** 10.1186/1546-0096-9-S1-O27

**Published:** 2011-09-14

**Authors:** S Habibi, J Ellis, H Strike, AV Ramanan

## Background

Prevalence of temporomandibular arthritis in JIA varies widely, reported rates ranging from 17- 87%. Untreated inflammation with joint destruction can lead to asymmetrical mandibular growth with jaw deviation, dental malocclusion and micrognathia. Intra-articular steroid injection for TMJ arthritis has been found to be effective. There are limited reports on the efficacy of ultrasound guided steroid injections of the TMJ’s in JIA.

## Aim

To assess the safety and efficacy of ultrasound guided corticosteroid injection, done by a paediatric rheumatologist, into the temporomandibular joints in children with JIA.

## Methods

Children with JIA presenting to rheumatology clinic assessed for TMJ arthritis. Triamcinolone hexacetonide injected in those with active arthritis assessed by MRI, using ultrasound guidance under general anaesthesia by a single paediatric rheumatologist trained in procedure. Efficacy and safety was assessed post-injection by patient guided symptoms and physical examination.

## Results

38 children (34 girls) with TMJ injection between Jan 2009–Jan 2011 studied. Mean age: 12.25±3.55years(range=5-18 years). Mean disease duration: 4.54±2.73 years (1.5-11.1years). Symptoms pre-injection: pain:17/38(44.7%), jaw deviation:14/38(36.8%), restricted jaw movement:13/38(34.2%), chewing dysfunction:7/38(18.4%), micrognathia:5(12.5%). Total 63 joints injected. Injection efficacious:58/63(92.06%) joints(Table 1). Stiffness was persistent in 2 children with both TMJ’s injected & 1 had persistent jaw deviation. Injection site scar in 1 child.

**Table 1:** Improvement in symptoms

Symptom	Improvement [n (%)]
Pain (17)	17 (100%)

Jaw deviation (14)	13 (92.8%)

Chewing dysfunction(7)	5 (71.4%)

## Conclusions

Ultrasound guided corticosteroid injection into the temporomandibular joint done by a paediatric rheumatologist trained in the procedure safe with a high rate of success.

